# Endothelial nitric oxide synthase intron 4a/b polymorphism in coronary artery disease in Thrace region of Turkey

**DOI:** 10.1080/13102818.2014.980030

**Published:** 2014-11-21

**Authors:** N. Sivri, A. Unlu, O. Palabiyik, M. Budak, Y. Kacmaz, K. Yalta, T. Sipahi

**Affiliations:** ^a^ Department of Cardiology, School of Medicine, Trakya University, Edirne, Turkey; ^b^ Department of Biophysics, School of Medicine, Trakya University, Edirne, Turkey

**Keywords:** coronary artery disease, endothelial nitric oxide synthase, polymorphism intron 4a/b

## Abstract

Coronary artery disease (CAD) is one of the frequent cardiovascular mortality causes in the world. Common risk factors explain only about half the risk of CAD. The healthy familial predisposition to CAD, combined with advances in genetic analysis, has led to a number of studies in recent years making an effort to identify the genetic factors that influence the risk. The approach taken by most studies was to examine the association of naturally occurring genetic polymorphisms in candidate genes with risk of or severity of CAD. Endothelial nitric oxide synthase (eNOS) is important for vascular and tissue protection and is found in endothelial cells that encompass the entire vasculature, including the vessels in the heart. Nitric oxide (NO) is produced in a catabolic reaction in the endothelial cells, neurons, glia and macrophages by nitric oxide synthase (NOS) isoenzymes. eNOS is a subgroup of this family of enzymes that catalyses the production of nitric oxide (NO) from L-arginine and oxygen, which leads to vascular relaxation by activating the guanylate cyclase. This finally induces smooth muscle relaxation. The aim of this study was to investigate the allelic frequency and the genotypic distribution of the variable number of tandem repeat 27 (27 VNTR) gene polymorphism in intron 4 of the eNOS (eNOS 4a/b) gene in Thrace region, to compare CAD patients with appropriate healthy controls and to correlate the genetic findings with CAD subtypes. The study group included 281 (153 subjects with CAD and 128 controls) patients. The eNOS polymorphism was identified with a polymerase chain reaction. Genotypes were defined as aa, ab and bb according to the presence of a and b alleles. In this case–control study, we found that there was sensible correlation between eNOS gene intron 4a/b VNTR polymorphism and the risk of CAD in Thrace region of Turkey. However, there was no major difference for the genotype distribution and the allelic frequency among the CAD subtypes. Further studies on the interaction of such genes are needed to clarify the association between eNOS 4a/b polymorphism and CAD patients.

## Introduction

Coronary artery disease (CAD) is the major cause of cardiovascular mortality worldwide. Among many traditional risk factors for CAD development, positive family history is now being considered as a significant risk factor. Atherosclerosis is the result of a defective endothelial function and an essential factor for the development of CAD.[1] Numerous studies support that the endothelial dysfunction plays a major role in the initiation and progression of atherogenesis.[2,3] Various factors including smoking, diet, aging and diseases affect the proper functioning of the endothelium.[4] Genetic predisposition also imposes a considerable risk for the development of atherosclerosis and cardiovascular diseases. The endothelium has essential role in maintaining vascular tone and blood pressure and that is largely mediated by nitric oxide (NO).[5] Other cardioprotective effects of NO include the inhibition of platelet aggregation, leukocyte adhesion and smooth muscle cell proliferation.[6,7] Therefore, the reduced bioavailability of NO is common to CAD, and defects in NO production and function correlate well with the incidence of CAD. NO is produced by the nitric oxide synthase (NOS) isoenzymes (endothelial, neuronal and cytokine-inducible) which are encoded by three distinct genes. NO is produced from L-arginine and oxygen in the endothelial cells, neurons, glia and macrophages. NO is the most powerful endogenous vasodilator agent. It can also inhibit the adhesion, aggregation and recruitment of platelets, promote vascular smooth muscle cell migration and growth, regulate some vessel–platelet interactions and limit the oxidation of atherogenic low-density lipoproteins.[8] Obvious evidence suggests that CAD is related to defects in the generation or action of NO. Different polymorphisms of the endothelial nitric oxide synthase (eNOS) gene have already been defined. Low NO release is known to predispose humans to atherosclerosis, hypertension, thrombosis and vasospasm. eNOS polymorphism is associated with reduced NO production and coronary artery spasm in different populations.[9–11] The evidence suggests that NO may inhibit several key steps in the atherosclerosis process and that an alteration of NO production within the vascular endothelium could contribute to pathogenesis of atherosclerosis.[12] The variable number of tandem repeat 27 (27 VNTR) polymorphism in intron 4 of the eNOS gene (eNOS 4a/b) and GT substitution in exon-7 in codon 298 are associated with an excess of risk of CAD.[13] Some previous studies revealed that NO concentrations were significantly higher in CAD patients than in controls, and, in some of them, there was an association between NO concentration and NOS3 gene polymorphism.[14] We aimed to investigate the 27 VNTR polymorphism in intron 4 of the eNOS gene in CAD in Thrace Region, because of higher CAD incidence in recent years.

## Materials and methods

A total of 153 subjects with CAD and 128 controls were enrolled in this study. The eNOS 4a/b was genotyped using a polymerase chain reaction (PCR) method. All the CAD patients were examined by the cardiologist and the diagnosis of CAD was established in accordance with the criteria of international classification. The control groups consisted of healthy volunteers. The history of hypertension, smoking habit and the existence of cardiovascular disease in the family of the CAD patients and controls was recorded. The study was approved by the local ethic committee and informed consent was obtained from the study cases.

### DNA isolation and PCR

**Figure 1.**
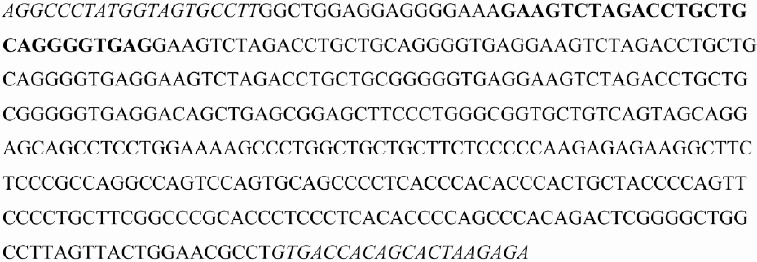
The sequencing of the region which contains eNOS 4a/b (27 VNTRs) polymorphism. Italic letters are used for the primer sequences and bold letters are used for 27-bp repeats which are deleted in the VNTR 4a polymorphism.

DNA was isolated from peripheral blood, collected into tubes containing ethylenediaminetetraacetic acid (EDTA) by eZNA (EaZy Nucleic Acid Isolation) blood DNA kits (Omega Bio-tek, Doraville, USA). eNOS 4a/b was identified using a PCR technique. eNOS intron 4a/b polymorphism was determined by PCR using oligonucleotide primers (sense: 5′-AGGCCCTATGGTAGTGCCTTT-3′; antisense: 5′-TCTCTTAGTGCTGTGGTCAC-3′; Figure 1) that flank the region of the 27 bp VNTR in intron 4. A genomic DNA was amplified by PCR in a total 25 μL of PCR mixture containing 200 ng of DNA, deoxynucleotide triphosphates (dNTPs; 0.2 mmol/L of each dNTP), 0.5 mmol of sense and anti-sense oligonucleotide primers, 1× Taq buffer and 1.25 U of Taq DNA polymerase. eNOS 4a/b gene polymorphism reactions contained 2.5 mmol/L MgCl_2_. All reagents for PCR amplification and gel electrophoresis were purchased from Fermentas Life Sciences (ELİPS, Istanbul, Turkey).

**Figure 2.**
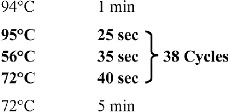
PCR procedure.

All other chemicals of the highest purity available were obtained from Sigma and Merck. DNA amplifications were performed with a Techne (TechGene) DNA Thermal Cycler. The thermocycling procedure consisted of initial denaturation at 95 °C for 25 s, annealing at 56 °C for 35 s and extension at 72 °C for 40 s (Figure 2).

**Figure 3.**
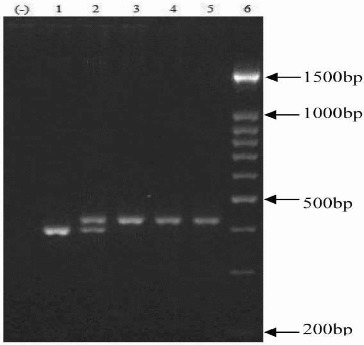
PCR products of eNOS VNTR gene polymorphism: aa genotype (394 bp; lane 1), ab genotype (394 bp and 421 bp; lane 2) and bb genotype (421 bp; samples 3, 4 and 5). Lane (−) is a negative control, and lane 6 is a size marker (O’Range Ruler 100 bp DNA ladder).

The PCR products were analysed using 2.5% agarose gel electrophoresis at 90 V for 1 h and visualized by ethidium bromide staining. The large allele eNOS 4b contains five tandem 27 bp repeats, and smaller allele eNOS 4a contains four repeats. The sizes of the PCR products were 393 and 420 bp for eNOS 4a and eNOS 4b alleles, respectively (Figure 3).

### Statistical analysis

All statistical analyses were performed with the SPSS 15.0 software. Data are presented as mean ± SD, median or per cent frequency. The frequencies of the genotype distribution (4a/a, 4a/b and 4b/b) between the patients and the controls were compared by χ^2^ test. Distribution of genotypes within groups was in accordance with the distribution predicted by the Hardy–Weinberg equilibrium model. Continuous variables among the genotypes were compared with the use of ANOVA and a *post hoc* Tukey test. Independent sample *t*-test was used to compare continuous variables between the patients and the controls. The odds ratio (OR) with a 95% confidence interval (CI) of the possible risk factor for CAD was evaluated by binary logistic regression analysis. Categorical data were compared by χ^2^ and Fisher's exact test where appropriate. The triglyceride pressure measurements significantly deviated from a normal distribution by Kolmogorov–Smirnov test. Therefore, log-transformed values were used for these parameters. Univariate and logistic regression analyses were used to evaluate the significant associations between the study parameters and the genotypes. All tests were two sided and a *P* value < 0.05 was considered as significant.

## Results and discussion

Our study included 281 subjects: 153 CAD patients and 128 controls (Table 1). All participants gave informed consent to examinations and genetic analysis of blood samples and the study was approved by the institutional ethics committee. There were significant age differences (*P* = 0.002); HDL (*P* < 0.001), LDL (*P* < 0.001), TG (*P* = 0.003) and TC (*P* < 0.001); and the frequencies of hypertension (*P* = 0.043); current smoker (*P* = 0.01) and family history of CAD (*P* = 0.029) between the CAD and control groups. The genotype frequencies were in agreement with those predicted by the Hardy–Weinberg equilibrium.

**Table 1.** Clinical characteristics of the control and CAD groups.

	Control group (*n* = 128)	CAD group (*n* = 153)	*P*
Age (years)	49.5 ± 5.4	53.0 ± 4.5	0.002
Sex (M/F)	53/75	59/94	Ns
Hypertension (%)	38.3	50.3	0.043
Current smoker (%)	29.7	49.0	0.01
Family history of CAD (%)	30.5	43.1	0.029
HDL (mg/ml)	39.2 ± 4.8	35.1 ± 7.3	0.000
LDL (mg/ml)	117.12 ± 20.3	136.16 ± 28.2	0.000
TG (mg/ml)	122.45 ± 66.2	148.5 ± 84.3	0.003
TC (mg/dl)	180.8 ± 42.5	199.9 ± 46.4	0.000

Note: HDL/LDL: high/low-density protein, TG: triglycerides, TC: total cholesterol, Ns: not significant.

Table 2 summarizes the clinical features and biochemical characteristics of the study groups according to the eNOS 4a/b genotypes. The significant effect of the eNOS gene intron 4ab VNTR polymorphism on plasma HDL, LDL and TC level in patients with CAD remained significant (*P* < 0.001) after adjustment of several potential confound factors.

**Table 2.** Clinical characteristics of controls and CAD.

Demographic and clinical characteristic of controls and CAD according to genotypes						
	Controls (*n* = 128)			CAD (*n* = 153)		
Variable	4a4a (*n* = 2)	4a4b (*n* = 21)	4b4b (*n* = 105)	4a4a (*n* = 4)	4a4b (*n* = 48)	4b4b (*n* = 101)
Hypertension (%)	0.8	6.3	31.3	2.0	18.3	30.1
Current smoker (%)	0.8	7.0	21.9	0.7	17.0	31.4
Family history of CAD (%)	0	4.7	10.2	0	10.5	26.8
Age (years)	45.0 ± 2	48.1 ± 6.2	49.5 ± 5.4	52.7 ± 5.1	52.6 ± 3.3	53.3 ± 5.0
Sex (M/F)	0/2	9/12	44/61	3/1	19/29	37/64
HDL (mg/ml)	40.5 ± 0.7	38.14 ± 5.1	39.4 ± 4.8	35.12 ± 8.12	35.26 ± 6.9	35.02 ± 101
LDL (mg/ml)	120.8 ± 18.5	119.8 ± 17.8	116.5 ± 20.9	114.7 ± 8.2	139.6 ± 27.7	135.3 ± 28.7
TG (mg/ml)	154.5 ± 126.5	93.9 ± 37.02	127.5 ± 68.7	176.2 ± 120.3	156.7 ± 98.7	143.5 ± 75.5
TC (mg/dl)	182.5 ± 7.7	178.4 ± 40.3	181.2 ± 43.5	191.2 ± 18.5	203.1 ± 50.2	198.8 ± 45.5

Note: HDL/LDL: high/low-density protein, TG: triglycerides, TC: total cholesterol.

Control and CAD groups’ genotypes and the risk of developing CAD are shown in Table 3. We discovered that aa homozygote genotype is more frequent in patients (2.6%) than in controls (1.6%), but this frequency is not related to the risk (*P* = 0.404). Patients carrying ab heterozygote genotype have a 2376-fold CAD risk (OR = 2.376, CI = 1.329–4.248, *P* = 0.004).

**Table 3.** Control and CAD groups’ genotypes and the risk of developing CAD.

eNOS 4a/b	Controls % (*n*)	CAD % (*n*)	OR	95% CI	*P*
bb	82.0 (105)	66.0 (101)	–	–	
ab	16.4 (21)	31.4 (48)	2.376	1.329–4.248	0.004
aa	1.6 (2)	2.6 (4)	2.079	0.373–11.602	0.404

Note: OR: odd's ratio, CI: confidence interval.

eNOS gene polymorphism may influence the functional activity of the enzyme and has modulating effects on atherogenesis. Previous studies have indicated phenotypic significance of eNOS 4a/b polymorphism in various conditions and its indirect effects on atherogenesis.[15–18] In addition, Wang et al. [20] reported that this polymorphism could influence transcriptional activity of eNOS gene. Tsukada et al. [15,16] showed that plasma NO level of healthy subjects with ‘a’ allele was significantly lower than in those without ‘a’ allele (*P* < 0.05).

eNOS gene polymorphism may have an influence on the functional activity of the enzyme, and, moreover, it has modulating effects on atherogenesis. Phenotypic significance of eNOS 4a/b polymorphism in various conditions and indirect effects on atherogenesis were shown in previous studies.[15–18] Furthermore, Wang et al. [20] reported that this polymorphism could affect transcriptional activity of eNOS gene. Tsukada et al. [15] indicated that plasma NO level of healthy subjects with ‘a’ allele was significantly lower than in those without ‘a’ allele (*P* < 0.05). Fatini et al. [21] showed that the 4a allele and the combined genotypes of ‘a’ allele with other eNOS gene polymorphisms were considerably associated with carotid atherosclerosis. In this study, a relatively high incidence of 4a allele and combined genotype of ‘a’ allele with another polymorphism (T-786C) was observed in a subset of patients with no traditional risk factors for atherogenesis (*n* = 30). This observation has been affirmed by Ichihara et al.[22] In Ichihara's study, it was determined that eNOS 4a allele was an independent risk factor for myocardial infarction, especially in patients lacking other conventional risk factors (*n* = 104). Hence, the harmful effects of eNOS 4a allele on atherosclerosis may appear independently from conventional risk factors. Asakimori et al. [23] reported that OR for carotid plaque positivity was increased by a factor of 3.72 in the presence of ‘a’ allele of intron 4 polymorphism in non-diabetic haemodialysis patients. Nevertheless, Lembo et al. [24] could not obtain any meaningful difference between the frequency of intron 4a/b polymorphism in hypertensive patients with and without carotid plaques.

Established cardiovascular risk factors including dyslipidaemia, smoking, hypertension and diabetes mellitus have been incorporated into algorithms for risk assessment in the general population,[25] but these characteristics do not fully explain the cardiovascular risk. There is substantial interest in the use of new biomarkers to identify individuals who are at risk for the development of cardiovascular disease and who could be targeted preventive measures.[5,26] Many individual biomarkers have been related to cardiovascular risk in ambulatory patients, including inflammatory markers and genetic polymorphism.

In the present study, we observed that eNOS 4a/b expression was increased in the CAD group compared to the control group. The results also exhibited that eNOS intron 4a/b 27 VNTR polymorphism was associated with CAD, and homozygote 4a4a genotype frequency was significantly higher in the CAD group compared with the control group. Furthermore, the results stated that heterozygote 4a4b genotype carriers have increased the level of eNOS expression compared with the same genotype in the control group which might have contribution to reduce the risk of CAD.

## Conclusion

In this case–control study, we found that there was a reasonable relation between eNOS gene intron 4a/b VNTR polymorphism and the risk of CAD. We also exhibited that eNOS intron 4b/4a 27 bp VNTR polymorphism was associated with CAD, and aa genotype frequency was significantly higher in CAD people in comparison with normal people. The limitation of our study was especially selected population, so that it does not represent common population but patients referred for CAD. This finding is potentially important; however, more detailed analyses are necessary to elucidate the associations of genetic abnormalities and CAD and its risk factors.
